# Chronic Immune Activation and CD4^+^ T Cell Lymphopenia in Healthy African Individuals: Perspectives for SARS-CoV-2 Vaccine Efficacy

**DOI:** 10.3389/fimmu.2021.693269

**Published:** 2021-06-17

**Authors:** Dawit Wolday, Francis M. Ndungu, Gloria P. Gómez-Pérez, Tobias F. Rinke de Wit

**Affiliations:** ^1^ Department of Medicine, Mekelle University College of Health Sciences, Mekelle, Ethiopia; ^2^ Department of Global Health, Kenyan Medical Research Institute (KEMRI) – Wellcome Research Programme, Nairobi, Kenya; ^3^ Amsterdam Institute of Global Health and Development, Department of Global Health, Amsterdam University, Amsterdam, Netherlands; ^4^ Joep-Lange Institute, Amsterdam, Netherlands

**Keywords:** Africa, chronic immune activation, SARS-CoV-2, vaccine, hyporesponsiveness, COVID-19, helminths

## Abstract

Chronic immune activation has been considered as the driving force for CD4^+^ T cell depletion in people infected with HIV-1. Interestingly, the normal immune profile of adult HIV-negative individuals living in Africa also exhibit chronic immune activation, reminiscent of that observed in HIV-1 infected individuals. It is characterized by increased levels of soluble immune activation markers, such as the cytokines interleukin (IL)-4, IL-10, TNF-α, and cellular activation markers including HLA-DR, CD-38, CCR5, coupled with reduced naïve and increased memory cells in CD4^+^ and CD8^+^ subsets. In addition, it is accompanied by low CD4^+^ T cell counts when compared to Europeans. There is also evidence that mononuclear cells from African infants secrete less innate cytokines than South and North Americans and Europeans *in vitro*. Chronic immune activation in Africans is linked to environmental factors such as parasitic infections and could be responsible for previously observed immune hypo-responsiveness to infections and vaccines. It is unclear whether the immunogenicity and effectiveness of anti-SARS-CoV-2 vaccines will also be reduced by similar mechanisms. A review of studies investigating this phenomenon is urgently required as they should inform the design and delivery for vaccines to be used in African populations.

## Introduction

CD4^+^ T cells play a pivotal role in the regulation of the immune system, protecting the host against various pathogens and autoimmunity (1). CD4^+^ T cells modulate the immune response by orchestrating responses of B-cells, CD8^+^ T cells and other components of the immune system. To do so, CD4^+^ T cells, also designated as T-helper (TH) cells, branch into five major subsets: TH1, TH2, TH17, follicular T helper (TFH) and regulatory T cells (Treg) CD4^+^ T cells (1). These different subsets of TH cells are differentiated by the expression of different lineage markers and transcription factors as well as the production of different cytokines. TH1 subsets express transcription factor T-bet, produce interferon (IFN)-γ, and they exert effector function against intracellular microorganisms, such as mycobacteria and viruses; TH2 subsets express transcription factor GATA-3, produce the cytokines interleukin (IL)-4, IL-5, IL-6, IL-13, and they exert effector function against extracellular organisms, such as helminths; TH17 subsets express transcription factor RORγT, produce IL-17, as well as IL-22, and they exert effector function against intracellular organisms, such as bacteria and fungi; TFH CD4^+^ T cells express transcription factor Bcl6, produce IL-21 and their effector function is to help B-cells produce immunoglobulins; Tregs express FoxP3 transcription factor, produce the cytokines IL-10 and transformation growth factor(TGF)-β, and their effector functions include immune homeostasis (1). Tregs propitiate an adequate immune response by helping in the recruitment of T cells, facilitating the removal of pathogens and preventing excessive tissue damage; hence they are critical in the prevention of autoimmunity and other forms of immune dysregulation (2). The differentiation of CD4^+^ T cells into the various subsets described above is highly dependent on the prevailing cytokine environment (1). Notably, dysregulation in CD4^+^ T cell immune responses may result in failure to protect the host from an infection or can lead to autoimmunity.

Previous reports showed that chronic immune activation is linked to the pathogenesis of CD4^+^ T cell lymphopenia in patients with HIV-1 infection (3). Moreover, the background immune profile of adult HIV-negative individuals living in Africa demonstrate a phenomenon of chronic immune activation, reminiscent of that observed in HIV-1 infected individuals. Here, we reviewed the potential influence of chronic immune activation or immune dysregulation and low CD4^+^ T cell count background against pathogens and vaccines on individuals residing in Africa. In addition, we discuss the potential role of altered immune responses that may impact on SARS-CoV-2 vaccine efficacy.

## Chronic Immune Dysregulation in Africans

Systemic persistent immune activation has been considered as the driving force of CD4^+^ T cell depletion in people infected with HIV-1 (3). More importantly, effective antiretroviral therapy (ART) and suppressed HIV-1 viremia, appears not to reduce the accompanying chronic immune activation in these patients (3). One of the mechanisms leading to systemic chronic immune activation in HIV-1 infected patients has been ascribed to HIV-driven dysbiosis of the gut microbiota and microbial translocation into the blood stream (4–7). Interestingly, the immune profile of adult HIV-negative individuals living in Africa exhibit chronic immune activation, reminiscent of that observed in HIV-1 infected individuals (8–17). In addition, chronic immune activation in these apparently healthy individuals of African origin is accompanied by significantly lower CD4^+^ T cell counts and CD4/CD8 ratio when compared to Europeans (8–23).

Such a skewed immune background profile of the African population may have a “double-edged sword” outcomes when the host encounters another pathogen or when administered a vaccine. Whereas chronic immune activation with skewed TH2 and Treg background may increase risk of infections, in particular for responses that depend on TH1 immune responses, it may provide protection against chronic inflammatory conditions, such as allergy or autoimmunity. In the same token, chronic immune activation may reduce the potential efficacy of vaccines, including those targeted against SARS-CoV-2 infection. The immune profile of apparently healthy and HIV-negative Africans is characterized by increased levels of soluble and cellular markers for chronic immune activation. Soluble immune activation markers consisted of elevated levels of IgE, IgG, placental isoferritin and p75 soluble tumor necrosis factor (TNF) receptor (8). In addition to increased eosinophilia, the expressions of HLA-DR, CD38, CD28, CCR5, Ki67 in CD4^+^ as well as CD8^+^ T cell subsets among Africans is significantly higher when compared to Europeans (8–17). Reduction in CD4^+^ and CD8^+^ naïve T cells, whereas increase in CD4^+^ and CD8^+^ memory T cells, as well as increase in CCR5 expression were observed in these population. The background profile is also characterized by predominant TH2 and Treg immune response (9), and marked increase in lymphocyte apoptosis (10). In addition, chronic immune activation in apparently healthy individuals residing in Africa is accompanied by CD4^+^ T cell counts that are significantly lower when compared to Europeans (8–23). For example, absolute CD4^+^ T cell count among adult healthy Ethiopians is around 700 cells/µL on average as compared to around 1,100 cells/µL among the Dutch population or Israelis (9–17).

## Underlying Causes of Immune Dysregulation in Africans

The reason for observed chronic immune activation, low CD4^+^ T cell count and inverted CD4/CD8 ratio in the African population remains poorly understood. Nonetheless, we noted that it is not genetic in origin, but something that is acquired during life (9, 15, 17). Earlier, we studied changes in T-cell receptor excision circles (TRECs), HLA-DR and CD31 expression on CD4^+^ T cells and T-cell telomere lengths with age in healthy Ethiopian and Dutch individuals. At birth, CD4^+^ naïve T cell numbers and TREC contents in Ethiopians were found to be comparable to those in Dutch neonates (17). At very young age, however, both CD4^+^ naïve T cell numbers and TREC contents fell dramatically in Ethiopians, but not in Dutch children. These differences between Ethiopian and Dutch individuals remained persistently into adulthood, because CD4^+^ naïve T cell numbers and TREC contents decreased at similar rates with age in Ethiopian and Dutch individuals (13, 17). In addition, telomere lengths in Ethiopian individuals tended to be shorter than the Dutch counterparts (17). Though the changes we observed in CD4^+^ T cell counts and their activation status occurred at earlier ages in life, as early as five years, adolescence and middle-aged adults, these changes appear to be reminiscent of immunosenescence (24). This state of dysregulated immune response associated with older age later during life is characterized by a chronic low-level inflammation (also known as “inflammaging”) and a decline of the immune system function (24).

These findings showing that immune activation increased with increasing age strengthen the notion that that external/environmental factors play significant role in inducing chronic immune activation (9). In addition, Ethiopian migrants to Israel who stayed longer period of time (> 5 years) indeed exhibited reversal in terms of increased CD4^+^ T cell counts and CD4/CD8 ratio as well as reduction in chronic immune activation (8, 10). Increased activation-induced lymphocyte apoptosis could be also one of the underlying mechanisms leading to the observed low CD4^+^ T cell among the Ethiopian population (10). In addition, tuberculosis (TB) patients have been shown to exhibit T cell immune activation (25), and hence latent TB, highly prevalent in Africa (26), may drive immune activation in this population. The role of viral infections, such as hepatitis C virus (HCV), cytomegalovirus and Epstein-Barr virus, in inducing immune activation in Africans remains unknown.

Among the environmental factors that drive immune activation in Africa is infection by parasites, in particular helminths. An estimated 2 billion of the world’s population is infected with helminth parasites (27). Chronic helminthic infections are often associated with the development of TH2-biased and alternatively activated macrophages (M2) and type 2 innate lymphoid cells (2, 28). These responses are accompanied with the induction of cytokines such as IL-4, IL-5, IL-13, and increased eosinophilia, IgE as well as goblet cell hyperplasia. Whereas the TH2-skewed immune responses are important in controlling helminthic infections, they are also considered to play an important role in the repair of tissue damage as a result of helminth infections (2, 28). Besides inducing TH2 immune responses, helminths also induce a strong regulatory networks, characterized by the induction of Tregs (2, 28). The induction of Tregs enhances survival and persistence of helminths within their host, and concomitantly may affect responses to heterologous infection, antigen, or vaccine. Indeed, several reports have demonstrated that infection by intestinal helminth is associated with chronic immune activation (8, 10, 12, 16, 29, 30). Interestingly, deworming resulted in reversal of immune activation, characterized by decreased eosinophilia, reduced expressions of HLA-DR, increased naïve cells and reduced memory cells (8, 10, 12, 16). Similar to infection with HIV-1, helminths also can cause microbial translocation (31–33), that may eventually lead to systemic chronic immune activation. Furthermore, profound CD4^+^ T lymphopenia and immune activation in the absence of HIV-1 infection among patients with malaria has been demonstrated (34, 35). The more severe form of *P. falciparum* resulted in significantly higher CD4^+^ T cell reduction or immune activation than the milder form of *P. vivax* (34, 35). Given the fact that CD4^+^ T cell count was determined from the peripheral blood, however, we assume that this was due to lymphocyte redistribution rather than direct CD4^+^ T cell death. We assume that macrophages engulf red blood cells laden with malarial parasites and migrate to the spleen or liver and TH1 cells are then redistributed from the periphery to these organs to activate the intracellular killing mechanism of the macrophage.

In addition to helminths, socio-demographic factors may influence CD4^+^ T cell counts. CD4^+^ T cell counts were independently and positively associated with female gender, cigarette smoking and khat (*Catha Edulis*) chewing (21). However, there is paucity of data with regard to the effect of diet on CD4^+^ T cell count differences among populations. We and others reported previously that body mass index was positively correlated with CD4^+^ T cell counts (21, 36). In addition, HIV-negative children with malnutrition from Uganda exhibited lower CD4% when compared to non-malnourished children (37). Increased exposure to ultraviolet light during summer has been reported to decrease CD4^+^ T cell counts among Europeans (38, 39). Overall, differences in CD4^+^ T cell counts between populations may be attributed to differences in diet, genetic, ethnicity, gender, residence, altitude, methods used to enumerate the cells and other yet unidentified factors.

## Effects of Chronic Immune Activation on Immune Response to Other Infections or Vaccines

Systemic chronic immune activation may potentially be detrimental to the host. Notably, T cell hyporesponsiveness induced by helminths may result in increased susceptibility to infections, or reduced responses to vaccines (**Table 1**). Several studies have demonstrated that co-infection with helminths is associated with increased susceptibility to heterologous infections. For example, concomitant infection with helminths in patients with TB results in a plethora of immune responses with unfavorable clinical outcomes (40–42). Helminths-driven immune modulation in these conditions included reduced CD3^+^, CD4^+^, CD8^+^, natural killer (NK), CD4^+^CD25^high^ and IFN-γ responses, but increased eosinophilia, Tregs, IL-4, IL-5 and IL-10 responses. Notably, individuals with latent TB infection co-infected with helminths had lower frequency of CD4^+^IFN-γ^+^ T cells and increased in CD4^+^FoxP3^+^ T cells (Tregs) compared to those without helminth co-infection (43). Although protective immunity against TB is considered to be dependent on cellular immune responses, mycobacteria-specific humoral immune responses have been proposed to play an important role in protection. However, co-infection with *Strongloides stercoralis* has been shown to reduce mycobacteria-specific B-cell responses (44). Unfavorable consequences include increased susceptibility to TB, persistence in *Mycobacterium tuberculosis* as well as more protracted TB disease course. In addition, studies conducting in experimental animal models demonstrated that helminth co-infection induced expression of arginase-1 by macrophages within the lung tissue resulting in enhanced inflammation and disease severity (45).

**Table 1:** Summary of the helminth-induced hyporesponsive immune responses to heterologous infections and vaccines.

Impact on target disease	Co-infection	Effects on immune system	Outcomes	References
**Infectious diseases:**				
TB	Helminths	↓ CD3^+^, CD4^+^, CD8^+^, NK, CD4^+^CD25^high^ T cell subsets	↑ susceptibility/ progression	(40–45)
		↑ Tregs	Persistent MTB infection	
		↓ IFN-γ	Protracted TB clinical course	
		↑ IL-10	↓ sputum smear positivity for acid fast bacilli	
		↑ IL-5	↑ lung inflammation	
		↑ eosinophilia		
		↓ mycobacteria-specific B-cell responses		
		↑ arginase-1-expressing macrophages in the lung, type 2 granulomas		
Hepatitis C virus	*S. mansoni*	↑ GrzB^+^ Tregs	↑ hepatitis C viral load, transaminases	(46, 47)
		↓ hepatitis C virus-induced antiviral immunity	↑ circulating levels of hepatitis C virus NS4 protein and extracellular-matrix deposition	
			↑ aggravated liver disease	
HIV-1	Helminths	↓ CD4^+^ T cell counts	↑ disease progression	(48–51)
		↑ CCR5 and CXCR4 expression in CD4^+^ T cells	↑ HIV-1 viral load	
			↑ HIV-1 acquisition	
**Vaccines:**				
Tetanus toxoid	*S. mansoni*	↓ tetanus toxoid-specific TH1 responses (IFN-γ)	↓ vaccine efficacy	(52)
		↑TH2 responses (IL-4)		
Malaria	Helminths	↓ lymphoproliferation	↓ vaccine efficacy	(53)
		↓ IFN-γ responses		
BCG	Helminths	↓ lymphoproliferation	↓ vaccine efficacy	(53, 54)
		↓ IFN-γ responses	↓ vaccine efficacy in prenatally sensitized children	
		↓ IFN-γ response among children sensitized *in utero*		
Hepatitis B	*S. mansoni*	↓ anti-hepatitis-B surface antibody titers	↓ vaccine efficacy	(55)
Cholera	Helminths	↓fecal and serum IgA immune responses to the B subunit of cholera toxin	↓vaccine efficacy	(56)
Pneumococcal	Helminth	↓ opsonization *S. pneumoniae*	↓ vaccine efficacy	(57)
H1N1 influenza A	Helminth	↓ anti-H1N1 antobody titers	↓ vaccine efficacy	(58)
		↑ IL-10-dependent type 1 Tregs		
Malaria vaccine candidate	*T. trichiura*	↓ antibody response to GMZ2 malaria vaccine candidate	↓ vaccine efficacy	(59)
HIV vaccine candidate	*S. mansoni*	↓ HIV-specific cellular responses	↓ vaccine efficacy	(60)
		↓ Env-specific antibody responses		
**Antihelminthic therapy:**				
BCG or TB disease + HIV	Helminth	↓ CD4^+^ FoxP3^+^ T cells (Tregs)	↓ disease severity	(43, 61–63)
		↑ MTB-specific TH1 immunity	↑ vaccine efficacy	
		↓ eosinophilia	↑ clinical improvement	
		↓ IL-10		
pRBC	Helminth	↑ proinflamatory cytokines	↑ vaccine efficacy	(61)
		↓ expression of CTLA-4 on CD4^+^ T cells		
Cholera vaccine	*A.lumbricoides*	↑ serum vibriocidal antibody titers	↑ vaccine efficacy	(63)
HIV-1	Helminths	↑ in CD4^+^ T cell counts	↓ HIV-1 viral load	(48, 49, 64, 65)
			↓ HIV disease progression	
HIV vaccine Candidate	*S. mansoni*	↑ HIV-specific cellular response	↑ vaccine efficacy	(60)

CTLA, cytotoxic T lymphocyte-associated antigen 4; IFN, interferon; IL, interleukin; pRBC, Plasmodium falciparum-infected red blood cells; TB, tuberculosis; MTB, Mycobacterium tuberculosis; TH, T helper cells; Tregs, regulatory T cells.

Several reports have also showed that co-infection with helminths can attenuate immune response to important viruses with negative outcomes. For example, HCV patients co-infected with the helminth *Schistosoma mansoni* showed significantly increased GrzB^+^ Treg response, indicating reduced HCV-induced TH1 and attenuated antiviral immunity (46, 47). In these patients, helminth co-infection led to aggravated HCV-related liver disease characterized by significantly elevated HCV load and transaminases when compared to patients infected with HCV only. Previous studies undertaken by us and others demonstrated that co-infection with helminths correlated with much lower CD4^+^ T cell counts and significantly higher HIV-1 viral load compared to those without helminth coinfection (48–50), and plasma HIV-1 viral load strongly correlated to the intensity of helminth infection (48). In addition, a recent systemic review reported that co-infection with schistosomes increased the risk of HIV-1 acquisition by 4-fold, through mechanisms involving increased expression of CCR5 and CXCR4 HIV-1 co-receptors on CD4^+^ T cells and cervical mucosa lesions (51).

Likewise, several earlier reports showed that helminth-induced chronic immune activation leads to a significant negative effect on vaccine efficacy. Whereas tetanus toxoid-specific TH1 (IFN-γ) immune responses were significantly attenuated in individuals with *S. mansoni* infection, TH2 (IL-2) responses were significantly increased (52). Children infected with helminths showed significant reduction in lymphoproliferative responses to *Plasmodium falciparum*-infected red blood cells (pRBC) and BCG compared to those without helminths (53). Notably, both lymphoproliferative and IFN-γ responses were significantly increased upon CD4^+^CD425^high^ T cell depletion, indicating that Tregs modulate helminth-driven immune responses to mycobacterial and malarial antigens. Children infected with the helminth *S. mansoni* when given hepatitis B vaccine showed significantly reduced hepatitis B virus surface antibody (anti-HBs) titers compared to helminth uninfected children (55). Similarly, patients with cholera co-infected with helminths had reduced fecal and serum IgA immune responses to the B subunit of cholera toxin (CTB) when compared to those without helminth co-infection (56). Interestingly, prenatal sensitization *in utero* has also been reported to reduce vaccine efficacy. Children of helminth-infected mothers showed significantly lower IFN-γ responses to the mycobacterial antigen – purified protein derivative (PPD) following vaccination with BCG compared to children of helminth uninfected mothers (54). Similarly, other investigators also demonstrated using animal experiments that helminths attenuated vaccine efficacy. Administration of pneumococcal vaccine to mice chronically infected with helminths was impaired due to failure to opsonize effectively *S. pneumonia* for killing by alveolar macrophages (57). In another recent study, helminth infection was reported to suppress the efficacy of vaccination against seasonal influenza (58). Helminth-infected mice had reduced quantity and neutralizing quality of antibody responses following vaccination with H1N1 influenza A virus, and attenuated vaccine efficacy was accompanied with increased levels of IL-10-dependent type 1 Tregs. Other investigators have reported similar reduced responses to vaccine candidates among individuals infected with helminths. A study (59) demonstrated that antibody responses to a blood stage malaria vaccine candidate GMZ2 was significantly lower in *Trichuris trichiura* infected children when compared to the antibody responses among parasite negative controls. Using an animal experimental model (60), it was shown that *S. mansoni* infected mice had significantly lower HIV-1-specific immune responses after prime-boost vaccination with DNA+MVA, or MVA+gp120 compared to uninfected control mice. In addition, gp140 Env-specific antibody responses were significantly in *S. mansoni* infected mice compared to controls.

Notably, anthelminthic treatment led to improved immune responses. For example, there was a decrease in the frequency of Treg cells accompanied with increased CD4^+^IFN-γ^+^ T cells after anthelmintic treatment of individuals with latent TB infection (43). In addition, cell-mediated immune responses to mycobacterial antigens following BCG immunization improved significantly among those who received anthelminthic therapy before vaccination when compared to controls (61–63). Deworming also can lead to improved antimalarial immune responses (61). Similarly, albendazole treatment of children infected with ascariasis enhanced the vibriocidal antibody response to live attenuated oral cholera vaccine (66). In addition, deworming resulted in increased CD4^+^ T cell counts, or reduced viral load in HIV-infected patients (48, 49, 64, 65), and increased HIV-1-specific cellular immune responses to HIV-1 vaccine candidate (60).

Exposure to specific microbial antigens has been shown to induce a sustained epigenetic alteration in innate immunity [reviewed in (67)]. Such alterations result in an enhanced immune response to a repeat challenge by the same antigen, or to heterologous one. This phenomenon is termed as “trained immunity”. The concept of trained immunity was originally discovered in studies involving BCG vaccine (67). The studies have provided evidence that BCG-induced trained immunity can protect against multitude of diseases, including respiratory infections and cancer. The concept of trained immunity is, however, in contrast to helminth-induced hyporesponsiveness described in the earlier sections of this review.

## Chronic Immune Activation in Africans and COVID-19

COVID-19 patients, like those infected with HIV-1, exhibit also lymphopenia (68). Will low baseline CD4^+^ T cell counts among African individuals increase risk of COVID-19 severity? Though this remains to be elucidated, we hypothesize that this is not the case. We base this on the observations we made showing that 74% of all SARS-CoV-2 infections are asymptomatic and only 4.4% present with severe COVID-19 (69). Furthermore, parasites indeed appear to protect against severe COVID-19 (70), by counteracting against excessive TH1-induced hyperinflammation (71).

Similarly, despite low baseline CD4^+^ T cell counts and background chronic immune activation, earlier studies we conducted in Ethiopia showed that disease progression among HIV-1 infected patients was not accelerated (72). Indeed, it was similar when compared to their counterparts from Dutch who had relatively higher baseline CD4^+^ T cell count and very little immune activation. The rate of average CD4^+^ T cell count decline, in the absence of antiretroviral therapy, was around 36 and 66 cells/year in the Ethiopians and the Dutch, respectively (72). The findings suggested that the rate of CD4^+^ T cell decline was the most important factor associated with disease progression rather than baseline CD4^+^ T cell count. The proportion of proliferating Ki67+ cells within the naïve and memory CD4^+^ T cell subsets were lower in HIV-infected Ethiopians compared to Dutch HIV-infected patients matched for CD4^+^ T-cell count (17). Thus, the slower CD4^+^ T cell decline in HIV-infected Ethiopians might be explained by lower levels of proliferation.

**Figure 1:**
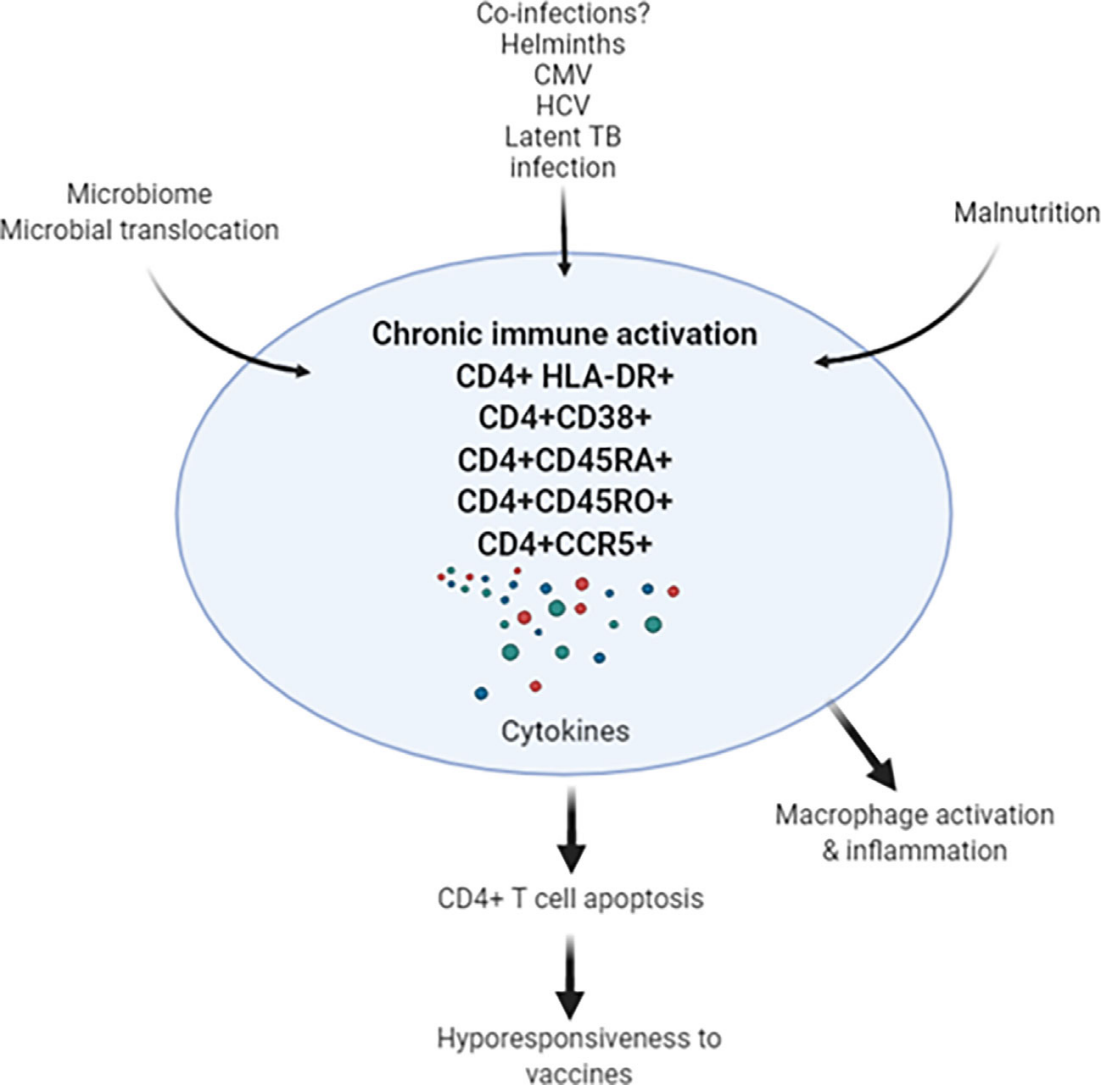
Chronic immune activation in apparently health Africans may be related to leaky gut, co-infections (such as helminths, CMV, HCV and latent TB infection) results in activation-induced CD4^+^ T cell apoptosis. In turn, depletion of CD4^+^ T cells may result in decreased response to SARS-CoV-2 vaccine in the African population. CMV, cytomegalovirus; HCV, hepatitis C virus; TB, tuberculosis.

Taken these observations, the important question is whether underlying chronic immune activation among Africans will impact on vaccine efficacies for SARS-CoV-2. Response to existing vaccines, such as BCG, yellow fever, rotavirus, polio, tetanus, influenza (73–76), as well as to candidate vaccines, such as for TB, malaria, Ebola and HIV-1, are lower when compared to responses in individuals in the northern hemisphere (77–80). Though the lower responses exhibited to the vaccines in the LMICs was observed irrespective of helminth status in the countries investigated, it is considered that co-infection with helminths is highly significant (81–83). Therefore, it is tempting to speculate that we might see similar reduced vaccine efficacies for SARS-COV-2 in LMICs (**Figure 1**). This notion is supported by a study that revealed Gabonese children living in rural areas with high incidence of helminth infection exhibited reduced anti-H1N1 and anti-B responses to a seasonal influenza vaccine compared to children living in semi-urban areas with lower incidence for helminth infection (83). Moreover, reduced vaccine efficacy has already been reported for various SARS-CoV-2 vaccines against the ‘South African variant’, B.1.351 (84). This reduction in efficacy was attributed to the specific B.1.351 mutations, particularly in the Spike-protein. However, the alternative explanation could be that the immune system of South African vaccine trial participants is skewed towards TH2, hyperactivated with low CD4^+^ T cells and therefore show less stimulation by SARS-CoV-2 vaccines.

In conclusion, studies are needed to evaluate the various factors that could potentially determine a reduced SARS-CoV-2 vaccine efficacy in Africa (85). These include both the effects of underlying immune activation status as described above as well as the influence of certain SARS-CoV-2 mutations. The current COVAX efforts in Africa will provide ample opportunities to evaluate these factors and findings should feed into COVAX vaccine distribution policies. Despite growing vaccine hesitancy in Africa, the roll-out of COVAX remains decisively important to avoid a situation of many years of persistence of significant SARS-CoV-2 outbreaks on the continent. Such events, particularly in immunocompromised individuals can lead to the emergence of new variants that could spread elsewhere in the world. The first evidence of such events is accumulating with a variant from Central Africa transmitting into Europe (86).

For all of the above reasons, we recommend that roll-out of COVAX and its potential reduced effectiveness due to different immune system backgrounds is proactively accompanied thorough information campaigns that involve African communities (87). Such campaigns should put into perspective the negative connotations that could be associated with the potential finding of generally reduced SARS-CoV-2 vaccine efficacy in African populations. Finally, we strongly recommend that highly prevalent infections, as the ones discussed in this manuscript, should be controlled for in future vaccine trials, not only for SARS-CoV-2 infection, to assess the real magnitude of the impact of these conditions on vaccine efficacy, to guide the design of African-tailored vaccines if needed. Eventually, an approach could emerge that involves the message: first deworm, then vaccinate.

## Author Contributions

DW and TR conceived the idea and drafted the review. All authors contributed to the article and approved the submitted version.

## Funding

This work was supported by the European and Developing Countries Clinical Trials Partnership (EDCTP) - European Commission (Project ID: RIA2020EF-2905), The Hague, The Netherlands and the Joep Lange Institute for Global Health and Development, Amsterdam, The Netherlands.

## Conflict of Interest

The authors declare that the research was conducted in the absence of any commercial or financial relationships that could be construed as a potential conflict of interest.
